# Enhancing Acoustic Emission Characteristics in Pipe-Like Structures with Gradient-Index Phononic Crystal Lens

**DOI:** 10.3390/ma14061552

**Published:** 2021-03-22

**Authors:** Gorkem Okudan, Hrishikesh Danawe, Lu Zhang, Didem Ozevin, Serife Tol

**Affiliations:** 1 Department of Civil, Materials, and Environmental Engineering, University of Illinois at Chicago, Chicago, IL 60607, USA; gokuda2@uic.edu; 2 Department of Mechanical Engineering, University of Michigan, Ann Arbor, MI 48109, USA; hgdanawe@umich.edu (H.D.); stol@umich.edu (S.T.); 3 College of Civil Engineering and Architecture, Guilin University of Technology, Guilin 541004, China; zhanglu@glut.edu.cn

**Keywords:** acoustic emission, attenuation, defects, GRIN lens, refraction, reciprocal property

## Abstract

Phononic crystals have the ability to manipulate the propagation of elastic waves in solids by generating unique dispersion characteristics. They can modify the conventional behavior of wave spreading in isotropic materials, known as attenuation, which negatively influences the ability of acoustic emission method to detect active defects in long-range, pipe-like structures. In this study, pipe geometry is reconfigured by adding gradient-index (GRIN) phononic crystal lens to improve the propagation distance of waves released by active defects such as crack growth and leak. The sensing element is designed to form a ring around the pipe circumference to capture the plane wave with the improved amplitude. The GRIN lens is designed by a special gradient-index profile with varying height stubs adhesively bonded to the pipe surface. The performance of GRIN lens for improving the amplitude of localized sources is demonstrated with finite element numerical model using multiphysics software. Experiments are conducted using pencil lead break simulating crack growth, as well as an orifice with pressured pipe simulating leak. The amplitude of the burst-type signal approximately doubles on average, validating the numerical findings. Hence, the axial distance between sensors can be increased proportionally in the passive sensing of defects in pipe-like geometries.

## 1. Introduction

Failure of pipelines may pose significant hazards leading to detrimental environmental and economical impacts. Since the beginning of this millennium, nearly 600 severe gas pipe incidents were reported with more than 200 fatalities [1]. Pipe failures are mainly caused by corrosion, manufacturing defects and external interferences leading to leakage or burst [2]. Disasters can be prevented by timely repairing after carrying out an effective inspection of defects. There is a multitude of pipe inspection techniques varying from visual inspection to smarter inspection techniques such as intelligent pigging. Visual inspection relies on natural senses, and is frequently inadequate as typically buried pipes will not be accessible. Fiber optic sensors are sensitive, but they are costly and difficult to implement for operational and buried pipes. The accuracy of radiographic methods is susceptible to temperature changes [3,4,5,6,7]. On the other hand, acoustic emission (AE) is a widely used passive nondestructive evaluation method that makes real-time and global inspection possible [8]. It does not only allow for the detection of defects, but also finding their location. The AE method is based on the detection of propagating elastic waves generated by active cracks, leaks or impact [9]. It was successfully implemented to pipelines by Juliano et al. as well as Ozevin et al. to find the location of leaks in pipe networks [10,11,12]. Furthermore, Mostafapour et al. used analytical modeling to locate leakage in a high pressure pipe, and then validated with experiments [13]. Current challenges of the AE method include the attenuation of propagating elastic waves in relatively long structures, the requirement of a large number of sensors for covering the entire range of pipeline, the interference of background noise and proper selection of wave velocity due to dispersive characteristics of pipes [14,15].

It is known that an AE source generates elastic waves within a hollow cylindrical structure, either in the form of burst signal caused by individual AE event such as crack growth and corrosion or continuous signal by repetitive AE events such as leak and friction. Elastic waves traveling in cylindrical structures have families of longitudinal, flexural and torsional wave modes which have different propagation characteristics. These wave modes have been utilized for the damage assessment of pipelines [16,17,18,19,20]. Higher order wave modes are known to have significant attenuation. Non-torsional fundamental wave modes such as the L(0,1) mode are also subjected to attenuation due to spreading and scattering by waves interacting with surface irregularities or change in pipe geometry [20]. The attenuation of elastic waves is a common problem in relatively long structures especially polymer-based and reinforced concrete pipes [21,22,23,24].

In this study, we investigate a new pipe configuration with a special refraction characteristic such that propagating linear elastic waves released from an AE source are converted into plane waves with the improved amplitude once the signal is reconstructed with an array of sensors at pipe circumference. Analogous to optics, mechanical waves have refractive characteristics as well, depending on the impedance of interface materials. A gradient-index phononic crystal lens (GRIN lens) is composed of scatterers [25] that are arranged periodically to manipulate elastic waves by changing the effective refractive properties of the base structure. Lin et al. proposed the first GRIN lens that can focus elastic waves at a known distance [26]. Since then, researchers has studied planar GRIN lens concept in plate-like structures in both macro [27,28,29,30] and micro [31,32] scales. More recently, we demonstrated a conformal GRIN lens for pipe-like structures to focus the guided wave energy at sensor locations at the focal points of the lens [33]. In this paper, we studied the reciprocal behavior of the conformal GRIN lens and leveraged it to convert the AE sources into plane waves for longer transmission of AE signals. We utilized a laboratory-scale pipe with a curved GRIN lens layer composed of cylindrical stubs made of steel with varying heights. We demonstrated the amplifying the elastic waves emitted by AE sources (burst or continuous) occurring near the focal point of lens numerically and experimentally. The extent of applications of phononic crystals goes beyond elastic wave focusing, with a variety of applications ranging from vibration reduction to acoustic waveguides and filters [34,35].

The outline of this paper is as follows—the GRIN lens design along with numerical model as well as the experimental configuration are presented in Section 2. Experimental results for the burst-type and continuous-type AE simulations are discussed in detail in Section 3. Conclusions and future work are discussed in Section 4.

## 2. Materials and Methods

### 2.1. GRIN Lens Design

GRIN lens is made of artificially engineered and graded materials with a variation of refractive index to modify the effective wave velocity. By special arrangement of graded materials, the energy of propagating elastic waves can be focused at a particular point for a particular frequency, which can be tuned by changing the spatial distribution and grading of intrusions. In this study, the GRIN lens is designed to convert the localized AE signal at the focal point into plane waves. The design is aimed to minimize the spreading effect of propagating elastic waves, specifically the longitudinal wave modes L(0,1) and L(0,2) at respective focusing locations without loss of information due to attenuation [36]. The effectiveness of the GRIN lens for the improvement of AE sensing in steel pipes is first verified numerically, and then validated experimentally. The specifications and the working principle of the proposed GRIN curved lens are described in Section 2.1.1. In Section 2.1.2, the performance of the GRIN lens is evaluated numerically.

#### 2.1.1. Specifications

**Figure 1:**
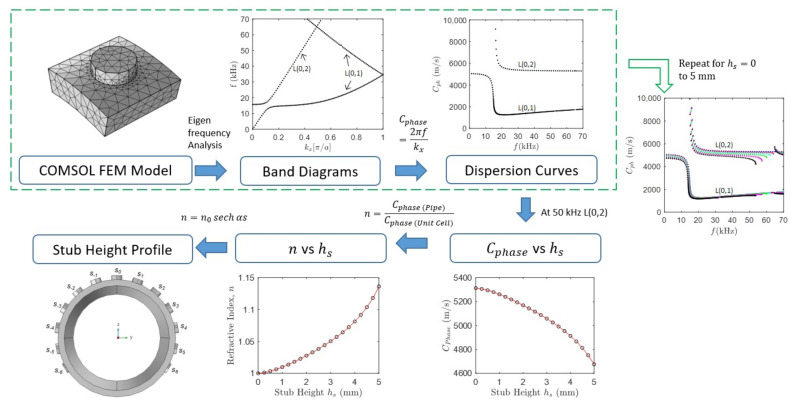
The flow chart of gradient-index (GRIN) phononic crystal lens design for steel pipe where *n* is the refractive index, $h_{s}$ is the stub height and $C_{phase}$ is the phase velocity of the propagating elastic wave mode, based on the design methodology in [33].

The GRIN lens design requires the identification of dispersion curves of individual unit cells to fit them into a shape of hyperbolic secant (HS) distribution of refractive index. Figure 1 illustrates the flow chart summarizing the design process of the GRIN lens. The first step is to identify unit cell size and diameter of the cylindrical stub, and obtain dispersion curves by eigenfrequency analysis of numerical unit cell models. This step is repeated for different stub heights ranging from 0 to 5 mm. Next, for a particular wave mode, at a certain frequency, the curve of phase velocity versus the stub height is obtained. Then, using the equation of refractive index [26], the relationship between refractive index and stub height is established. In the last step, the optimal stub height profile of the GRIN lens is obtained which corresponds to the hyperbolic secant distribution of refractive index resulting in focusing of the considered wave mode at the focal point of the lens.

**Figure 2:**
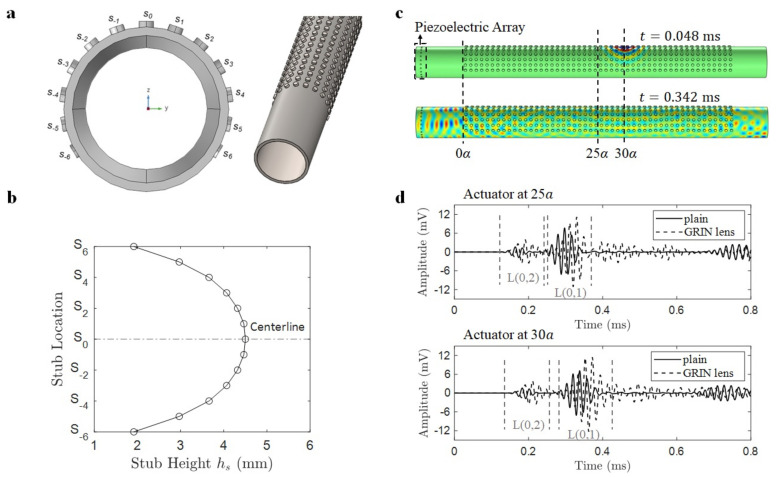
(**a**) Steel pipe with GRIN lens showing distribution of cylindrical steel stubs over pipe surface. (**b**) stub height profile designed using GRIN theory. (**c**) instantaneous radial velocity field showing reciprocal action of the lens (i.e., point source turning into plane wave) for piezo actuator located at 30a near focal point of L(0,1) mode. (**d**) amplification of wave modes with GRIN lens showing plane wave formation for two actuator positions of 25a and 30a.

The GRIN lens design is composed of cylindrical steel stubs of varying heights $\left(h_{s}\right)$ attached to outer surface of a steel pipe with a diameter of 114.3 mm and wall thickness of 6 mm. Figure 2a shows the arrangement of stubs around the pipe circumference along with a steel pipe integrated with GRIN lens. The lens aperture is chosen to be 13 unit cells-wide with the highest stub at the centerline of the lens and 6 stubs of decreasing heights on either side of the centerline. Each stub forms a unit cell with the axial and angular spacing between the stubs as the unit cell length *a* of 20 mm.

The stub heights follow a special profile as shown in Figure 2b, which results in HS distribution of refractive index for the longitudinal L(0,2) mode at a design frequency of 50 kHz [33]. The design is based on the gradient-index optics theory [37] where a hyperbolic secant distribution of refractive index within a GRIN lens results in focusing of optical waves at the lens centerline. The HS profile for refractive index (*n*) is given by:

(1) $$n=n_{0}sech\left(\alpha s\right),$$

where $n_{0}$ is the refractive index at the lens centerline, α is the gradient coefficient and *s* is the location of unit cell in the direction transverse to the lens centerline. With hyperbolic secant distribution of refractive index, the first focal point for wave focusing is obtained at π/2α. Thus, the designed stub height profile results in the first focal point of L(0,2) mode at a distance of 24.54a with a gradient coefficient of 0.064/α. Using the same design we found out the refractive index distribution for the longitudinal L(0,1) mode. The best HS fit to this distribution has a gradient coefficient of 0.0535/α resulting in the first focal point of L(0,1) mode at 29.36a. The pipe integrated with GRIN lens results in bending of plane wave towards the lens centerline because of the highest refractive index (due to the highest stub height) at that location and it eventually gets focused at the focal point predicted using the GRIN theory as presented in [33]. The waves bend from the region of lower refractive index (with lower stub heights) towards the region of higher refractive index (with higher stub heights) as per Snell’s law. The reciprocal phenomenon where a point source at a focal point of the lens turns into a plane wave after exiting the lens region is shown before [38]. The reciprocal action of the lens is further verified through numerical simulations.

#### 2.1.2. Numerical Verification

In order to verify the reciprocal action of GRIN lens, we performed time domain finite element numerical simulations using COMSOL Multiphysics software. A 1.5 m long steel pipe with a diameter of 114.3 mm and wall thickness of 6 mm was integrated with a 50 unit cells-long GRIN lens. An array of 24 equally spaced piezoelectric discs of 0.4 mm thickness and 5 mm diameter was attached at one end of the pipe to detect the plane wave. A similar piezoelectric actuator was attached near the first focal points of L(0,2) and L(0,1) modes at 25a and 30a, respectively, which would act as a point source. A first order tetrahedral mesh was created with the mesh size resulting in 12 elements within the smallest wavelength (of L(0,1) mode at 50 kHz) and time stepping was fixed according to Courant-Friedrichs-Lewy number of 0.2 for optimal wave solution. A half-pipe model with symmetry boundary conditions was used (to reduce computations) along with low-reflecting boundary conditions at the pipe ends (to minimize wave reflections). The piezoelectric actuator was excited using a 4-cycle sine burst excitation at 50 kHz with a Gaussian window simulating as a point source. The simulations were performed over a time window of 800 μs for actuator positions of 25a and 30a. For example, the time domain snapshots of instantaneous radial velocity field with actuator at 30a is shown in Figure 2c. The snapshots show that point source at the very beginning (t = 48.67 μs) turning into a plane wave as it exits the lens (t = 342.67 μs). The plane wave formation was further verified by the sensor readings of the piezoelectric array attached near the pipe end. The cumulative signal obtained by adding the readings of piezoelectric sensors is shown in Figure 2d for two source positions, and it is compared for conventional pipe versus the pipe with GRIN lens. The signal obtained with GRIN lens is amplified because the wavefront arrives simultaneously near the sensors in the piezo array resulting in constructive interference showing plane wave arrival. The wave packets are identified in the figure as L(0,2) and L(0,1) wave modes due to their different arrival times resulting from different wave speeds for these two modes. The amplification factors obtained for the excitation position of 25a are 2.39 and 1.45 for L(0,2) and L(0,1) modes, respectively. Similarly, the amplification factors obtained for the excitation position of 30a are 2.40 and 1.60 for L(0,2) and L(0,1), respectively. Thus, the numerical results verify the reciprocal effect of GRIN lens turning a point source into a plane wave with the amplified amplitude.

### 2.2. Experimental Design

For the experiments, a prototype steel pipe with a length of 1520 mm, a thickness of 6 mm, and an outer diameter of 114.3 mm was built in the laboratory. This is a typical pipe used in the oil and gas industry. The GRIN lens consisted of 650 cylindrical stubs made up of steel. Each stub had a diameter of 10 mm. Based on the refractive index profile, the stub heights were selected as 4.50 mm, 4.46 mm, 4.32 mm, 4.07 mm, 3.66 mm, 2.97 mm and 1.92 mm. The unit cell size, *a*, of the stubs was selected as 20 mm. Conforming to this design, the GRIN lens layer was built in the laboratory and attached to the pipe using adhesive.

**Figure 3:**
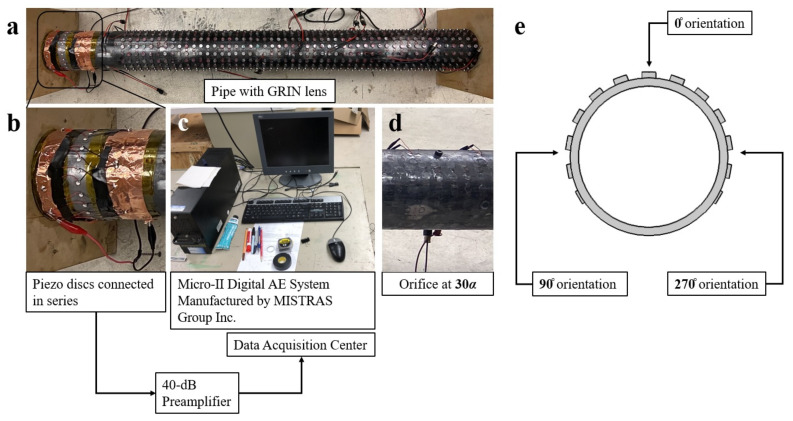
(**a**) Experimental configuration and schematic: pipe configuration with GRIN phononic crystal lens layer, (**b**) close-up view of piezoelectric discs connected in series, (**c**) the data acquisition system, (**d**) the orifice that was used in leak simulations and located at 30a, (**e**) cross-section of the pipe with GRIN lens illustrating the locations of pencil lead break tests performed at focal points.

An array of transducers made up of 24 equally spaced piezoelectric discs manufactured by Steminc was attached around the pipe circumference using adhesive. The thickness of each piezoelectric disc is 0.4 mm and the diameter is 5 mm. The piezoelectric discs connected in series were used as a receiver to detect propagating elastic waves generated by an AE source. The received AE signals were first amplified by 40 dB pre-amplifer, and then recorded with PCI-8 data acquisition system manufactured by MISTRAS Group, Inc (Princeton Junction, NJ, USA) The data collection variables were a sampling rate of 1 MHz and a digital filter of 20–200 kHz. Figure 3 illustrates the experimental configuration and schematic.

Two types of AE sources were generated. Initially, an AE source caused by instantaneous events such as crack growth or impact was simulated using pencil lead break (PLB) tests using 0.5-mm pencil lead conforming to ASTM standard [39]. PLB tests were performed along the centerline of the GRIN lens with 5a increments to see the focusing behavior. Moreover, at the predetermined 25a and 30a focal points, presented in the previous subsection, they were carried out at 90° and 270° orientations as well. A second sensor was attached next to each PLB simulation to act as the trigger channel so that temporal information about the arrival times of the wave modes could be obtained.

The AE phenomenon was also simulated by leakage from an orifice installed to the pipe using a threaded bolt shown on Figure 3. The orifice size was 1.27 mm. For the GRIN lens case, the stubs were attached on the pipe such that the orifice would be at the 30a focal point. The pipe was pressurized with air at 10 (68.95), 20 (137.90) and 30 (206.85) psi (kPa) internal pressure levels. The experiments were conducted for with and without GRIN lens.

## 3. Results and Discussion

### 3.1. Simulations Using Pencil Lead Break

**Figure 4:**
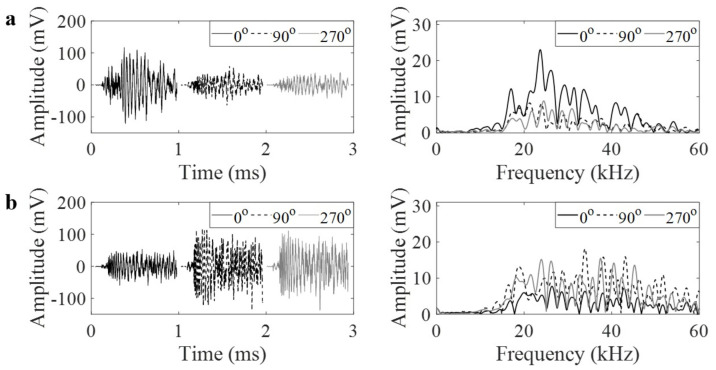
(**a**) Pencil lead break (PLB) waveforms in time and frequency domains at 0°, 90° and 270° at 25a position with lens, and (**b**) without lens.

**Figure 5:**
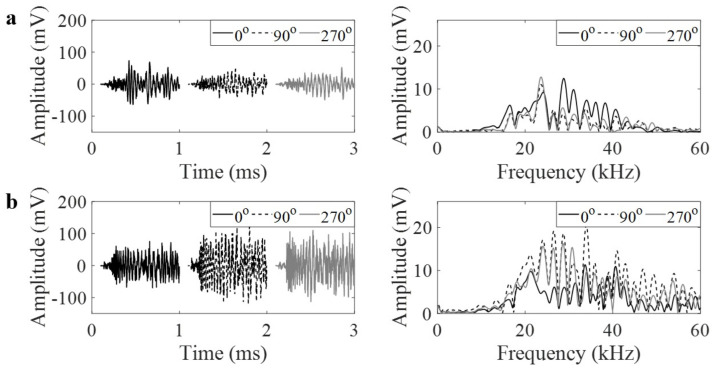
(**a**) PLB waveforms in time and frequency domains at 0°, 90° and 270° at 30a position with lens, and (**b**) without lens.

PLB generates a burst-type signal that is defined as the emission caused by an individual event occurring within a material. PLB waveforms in the time and frequency domains are shown for 25a and 30a focal locations in Figure 4 and Figure 5, respectively. The time domain waveforms were stacked next to other to present side-to-side comparison of their amplitudes in Figure 4. Each figure indicates the waveforms for the two cases: (a) with GRIN lens and (b) without GRIN lens. Each figure also represents how the waveforms vary with respect to the 0°, 90° or 270° orientations around the pipe circumference. As the GRIN lens is expected to focus the plane wave at a focal point, an increase in the amplitudes can be observed at 0° orientation. The frequency contents of AE signals at all degrees have similar characteristics when there is no lens. Once the lens is added to the pipe, the amplitude of AE signal near 30 kHz shows an increase at 0° as compared to 90° and 270°. At the 25a focal point and 30 kHz frequency, the AE amplitude increases from 6.2 mV to 13.3 mV. Similarly at the 30a focal point, it increases from 6.1 mV to 12.5 mV. Thus, in the configuration with the GRIN lens, the AE amplitude more than doubles at the design frequency of GRIN lens similar to numerical results.

### 3.2. Attenuation Curves at the Focal Line

**Figure 6:**
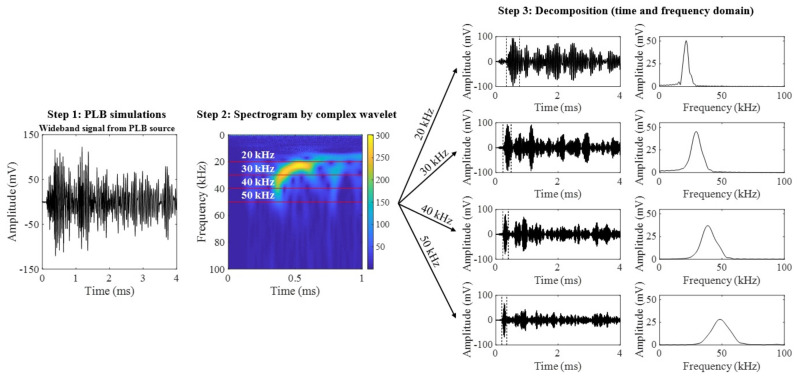
Wavelet decomposition of PLB waveform.

Since PLB generates a wideband signal consisting of waveforms of several frequencies, the AE signals were decomposed into components at 20, 30, 40 and 50 kHz using Continuous Wavelet Transform (CWT) with a “Morlet” mother wavelet [40]. Figure 6 indicates the steps taken for the wavelet analysis. The spectrogram in Step 2 was generated for each wideband PLB signal using CWT. The complex spectrogram was decomposed into the harmonics as shown in Step 3, by extracting lines at the desired frequencies. L(0,1) and L(0,2) wave modes can be identified from each decomposed signal based on the arrival times. The frequency spectrum shows the dominant frequency in the first wave envelope of time frequency signal.

**Figure 7:**
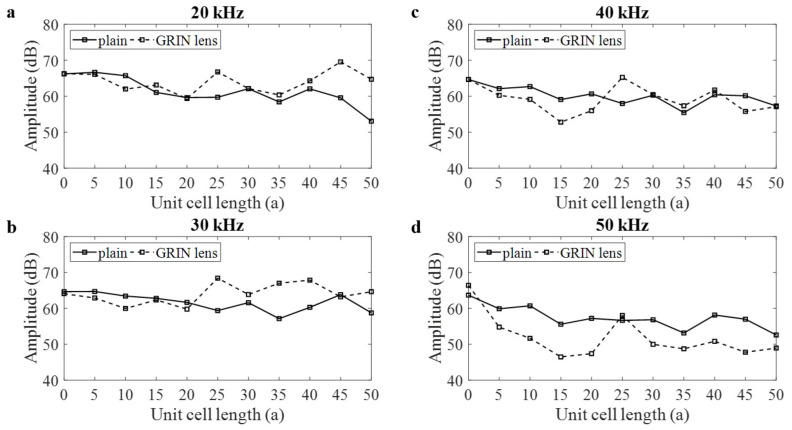
(**a**) Attenuation curves at 0° with and without lens for 20 kHz, (**b**) 30 kHz, (**c**) 40 kHz, (**d**) 50 kHz.

The amplitude of each decomposed signal was extracted along the centerline of the lens layer and plotted as shown in Figure 7. Voltage signal was converted into dB by taking 1 μV as the reference and subtracting 40 dB gain. For the plain pipe case, attenuation can be observed for all the frequencies. However, for the case with the GRIN lens, the attenuation curve starts to rise when it reaches 25a focal point, except for 50 kHz. For the case of 30 kHz frequency, the AE amplitude increases in the spatial distance of 25a to 40a, which can be considered as the effective area of GRIN lens to amplify AE signal. As it can be inferred from this figure, at 25a, the amplitude increases from 60 dB to 67 dB for 20 kHz. It increases from 59 dB to 68 dB for 30 kHz. Lastly, it increases from 58 dB to 65 dB for 40 kHz. At 30a, the amplitude increases only from 62 dB to 64 dB for 30 kHz. For 50 kHz, the amplitudes diminish with the presence of GRIN lens. A band gap occurs near 50 kHz for the maximum height stub, which causes the decay in amplitude at 50 kHz. This is due to the fact that the presence of adhesive layer slightly modifies the dispersion characteristics [41].

### 3.3. Detecting Leak Using GRIN-AE

**Figure 8:**
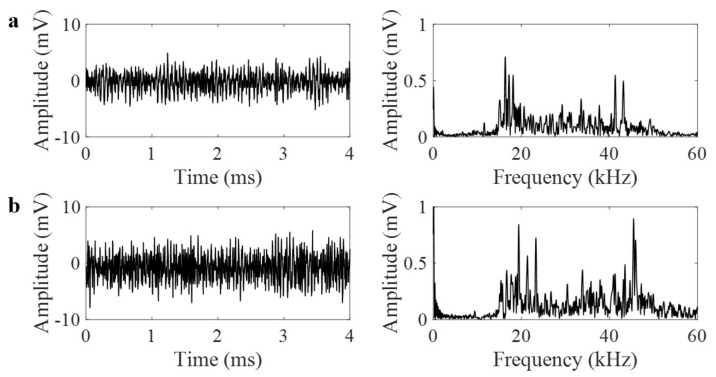
(**a**) Leak waveforms at 10 psi internal pressure in time and frequency domains with lens, (**b**) without lens.

**Figure 9:**
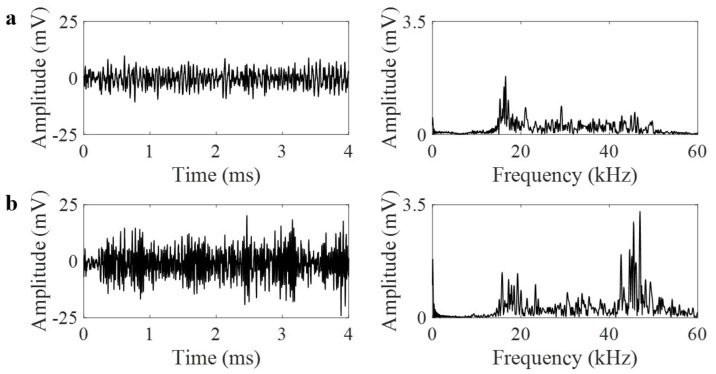
(**a**) Leak waveforms at 20 psi internal pressure in time and frequency domains with lens, (**b**) without lens.

**Figure 10:**
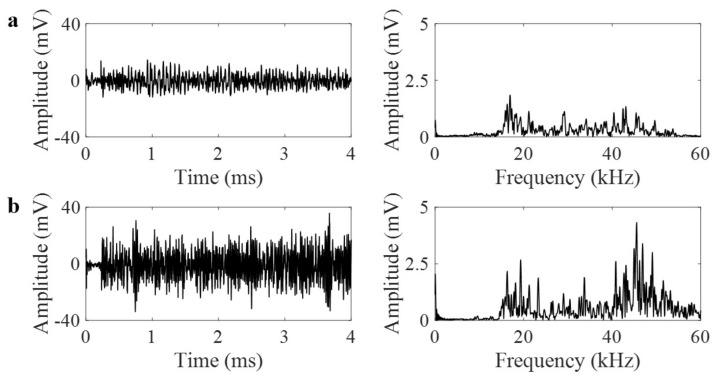
(**a**) Leak waveforms at 30 psi internal pressure in time and frequency domains with lens, (**b**) without lens.

Leak released from through-thickness hole in a pressurized pipe causes turbulent flow that is defined as the AE source. As it is a continuous source, it generates multiple burst-like signals that are added to form a continuous AE signal. As compared to burst-like AE signals that have well-defined rise-time and decay-time, leak signals have complex nature. Examples of leak signals obtained from three different internal pressures and with/without lens are shown in Figure 8, Figure 9 and Figure 10. All the waveforms and their frequency spectra are plotted in the same scale. Amplitude of leak signal increases with increasing internal pressure. Using the AE signals recorded from the pipe with lens shown in Figure 8a, Figure 9a, Figure 10a, the AE amplitudes for 10 psi, 20 psi and 30 psi internal pressure are 5.2, 10.7, and 14.3 mV, respectively. Using the AE signals recorded from the plain pipe shown in Figure 8b, Figure 9b, Figure 10b, the AE amplitudes for 10 psi, 20 psi and 30 psi internal pressure are 7.9, 24.1, and 35.7 mV, respectively. In general, the frequency spectrum of the pipe with GRIN lens focuses to the frequency range of 10–30 kHz where theoretical signal amplification is observed. However, as plain pipe has wide-band spectrum, the signal amplitude is slightly higher.

**Figure 11:**
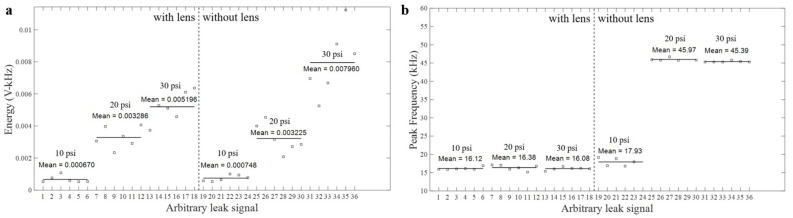
(**a**) Spectral energy of six selected acoustic emission (AE) signals for each pressure and pipe state in the frequency range of 10–30 kHz, (**b**) peak frequencies of six selected AE signals for each pressure and pipe state.

The energy in the frequency range of 10–30 kHz was extracted from the frequency spectra of six arbitrarily selected leak signals for three pressure levels and pipe states. Figure 11a shows that while there is a slight amplification observed for the pipe with GRIN lens at 20 psi pressure, they slightly decrease for 10 psi and 30 psi cases. This is attributed to the fact that GRIN lens is positioned only on one side of the orifice; therefore, theoretical amplification has not been observed. The presence of internal pressure may have changed the dispersion characteristics and the location of focal point. On the other hand, GRIN lens focuses the energy into a narrow frequency range as shown in Figure 11b. As compared to the plain pipe, the pipe with GRIN lens has similar peak frequency independent from leak rate. Well-focused dispersion characteristics assist in source localization as wave velocity in pipes is frequency dependent [11]. By designing pipe with a unique transfer function, source localization error due to frequency-dependent velocity can be minimized.

## 4. Conclusions

In this paper, a new pipe configuration with mechanical GRIN lens was introduced to reduce the attenuation of AE signal due to wave spreading in conventional pipes. With the reconfigured pipe geometry, the effective dynamic properties of pipe were altered in such a way that the attenuation of propagating elastic waves was overcome at certain regions. New pipe configuration improves the wave propagation distance, increases the spacing between AE sensors, and reduces the overall cost of structural health monitoring system. Additionally, the frequency response characteristics of pipe with GRIN lens shows a highly narrowband frequency, which reduces error in the source localization by selecting a proper wave velocity of dominating frequency. Numerical and experimental results show that the amplitude of burst-type AE signal approximately doubles, which means that the distance between AE sensors can be increased proportionally. The detectability of burst-type AE signals released by crack growth, corrosion and localized impact in pipelines can be enhanced by adding GRIN lens to pipe structure as the amplitude of propagating elastic waves released from a localized source is increased with GRIN lens, which brings the AE amplitude of minimum detectable defect energy above threshold. In future work, the influences of internal pressure and earth pressure for buried pipes to GRIN lens design will be investigated. Additive manufactured pipe design with the embedded GRIN lens into the manufacturing process will be explored.

## Data Availability

The data can be provided upon request from the corresponding author.

## Abbreviations

The following abbreviations are used in this manuscript:

AE	Acoustic Emission
GRIN	Gradient-Index
HS	Hyperbolic Secant
PLB	Pencil Lead Break
CWT	Continuous Wavelet Transform
